# The *Ustilago maydis* Effector Pep1 Suppresses Plant Immunity by Inhibition of Host Peroxidase Activity

**DOI:** 10.1371/journal.ppat.1002684

**Published:** 2012-05-10

**Authors:** Christoph Hemetsberger, Christian Herrberger, Bernd Zechmann, Morten Hillmer, Gunther Doehlemann

**Affiliations:** 1 Max Planck Institute for Terrestrial Microbiology, Marburg, Germany; 2 Institute of Plant Sciences, Karl-Franzens University of Graz, Graz, Austria; Purdue University, United States of America

## Abstract

The corn smut *Ustilago maydis* establishes a biotrophic interaction with its host plant maize. This interaction requires efficient suppression of plant immune responses, which is attributed to secreted effector proteins. Previously we identified Pep1 (Protein essential during penetration-1) as a secreted effector with an essential role for *U. maydis* virulence. *pep1 *deletion mutants induce strong defense responses leading to an early block in pathogenic development of the fungus. Using cytological and functional assays we show that Pep1 functions as an inhibitor of plant peroxidases. At sites of Δ*pep1* mutant penetrations, H_2_O_2_ strongly accumulated in the cell walls, coinciding with a transcriptional induction of the secreted maize peroxidase POX12. Pep1 protein effectively inhibited the peroxidase driven oxidative burst and thereby suppresses the early immune responses of maize. Moreover, Pep1 directly inhibits peroxidases *in vitro* in a concentration-dependent manner. Using fluorescence complementation assays, we observed a direct interaction of Pep1 and the maize peroxidase POX12 *in vivo*. Functional relevance of this interaction was demonstrated by partial complementation of the Δ*pep1* mutant defect by virus induced gene silencing of maize POX12. We conclude that Pep1 acts as a potent suppressor of early plant defenses by inhibition of peroxidase activity. Thus, it represents a novel strategy for establishing a biotrophic interaction.

## Author Summary

The maize pathogen *U. maydis* establishes a biotrophic interaction with its host plant and causes the formation of plant tumors. The *U. maydis* infection is initiated by a direct penetration of the plant epidermis and relies on living plant tissue. Therefore, suppression of the host immune system is essential for successful infection. Previously we identified the secreted effector Pep1, which is essential for *U. maydis* pathogenicity. *pep1* deletion mutants are blocked by host defense responses immediately upon penetration. In the present study we identified the molecular function of Pep1 and explain its crucial role for fungal virulence. We found that Pep1 inhibits the plant oxidative burst, which is characterized by the accumulation of reactive oxygen species (ROS) such as hydrogen peroxide. A conserved component of the plant ROS generating system are peroxidases. We could show that Pep1 directly inhibits plant peroxidases. One specific maize peroxidase (POX12), which was strongly induced by infection of the *pep1* deletion, directly interacts with POX12 *in vivo.* Moreover, POX12 silenced plants are penetrated by the *pep1* deletion mutant, indicating functional relevance of the Pep1-POX12 interaction. Together, these findings show that Pep1 directly interferes with the ROS-generating system of the host plant to suppress immune responses.

## Introduction

The basidiomycete smut fungus *Ustilago maydis* establishes a biotrophic interaction with its host plant maize which leads to the formation of plant tumors on all aerial parts of the host plant [1], [2]. After penetration of the leaf surface, pathogenic *U. maydis* hyphae proliferate inside host cells that stay alive and do not show any obvious defense responses [3]. Prior to establishment of biotrophy, *U. maydis* infection causes a transient defense response [1], [4]. This induction is most likely triggered by recognition of conserved pathogen-associated molecular patterns (PAMPs) through the maize immune system. With the onset of biotrophy 24 hours post infection (hpi), defense gene expression is attenuated. In line with the model of necrotrophic pathogens inducing primarily SA-dependent cell death responses including expression of defense genes like PR1 [5], biotrophic pathogens like *U. maydis* mainly induce the antagonistic JA and ethylene responses during compatible interactions [4], [6], [7].

Reactive oxygen species (ROS) are key molecules in plant defense [8]–[10]. The production of ROS is a hallmark of successful recognition of a pathogen and results in activation of plant defense responses, including oxidative burst, papilla formation, hypersensitive response (HR) and expression of PR genes [8], [11]–[14]. ROS can directly act toxic at the site of infection or function indirectly as second messengers. The origin of ROS in plant defense is largely attributed to two major sources: membrane bound NADPH-oxidases and apoplastic/cell-wall associated peroxidases (POX) [13], [15]–[17]. POX catalyze dehydrogenation of various phenolic and endiolic substrates by hydrogen peroxide (H_2_O_2_), resulting e.g. in the synthesis of lignin, suberin and the decomposition of IAA [8], [18], [19]. In addition, POX can exhibit oxidase activity, mediating the reduction of O_2_ to superoxide (O_2_^•−^) and H_2_O_2_ by substrates such as NADH or dihydroxyfumarate [8], [18]. Moreover, *in vitro* studies of horseradish peroxidase showed the generation of hydroxyl radicals (^•^OH) from reduction of hydrogen peroxide [18], [20].

The secretion of effector proteins by the pathogen that interact with targets of the host cell is a crucial aspect for the establishment of biotrophy. Effectors may mask the pathogen from recognition by the host immune system. For example, the LysM effector Ecp6 from *Cladosporium fulvum* sequesters chitin oligomeres originating from the fungal cell wall and therefore prevents PAMP-triggered immunity [21]. When suppressing an already triggered plant immune response, fungal effectors can either directly interact with a defense related protein or inhibit signaling pathways leading to defense responses. A direct inhibition of host defense proteins was shown for *Cladosporium fulvum* Avr2 that binds the host protease RCR3 and PIP2 to suppress host immunity [22]–[24]. On the other hand, the *Pseudomonas syringae* effector AvrPto blocks intracellular downstream signaling cascades by interfering with the flg22-triggered receptor FLS2 [25]. The effector protein AvrPiz-t, which is secreted by pathogenic hyphae of the hemibiotrophic ascomycete *Magnaporthe oryzae* suppresses BAX-induced programmed cell death in tobacco leaves [26].

In *U. maydis*, several gene clusters encoding putative effector proteins have been identified [27], [28]. However, the modes of action of these clustered effectors and the way they contribute to fungal virulence still remain elusive. Recently, the secreted effector Pit2 has been identified as an essential virulence factor of *U. maydis* [29]. The *pit2* gene resides in a small cluster next to *pit1*, which encodes a transmembrane protein. Both Pit1 and Pit2 are required for *U. maydis* induced tumor formation but the function of these proteins in the host plant still remains unclear [29]. A single effector of *U. maydis* that is essential for the establishment of biotrophy is Pep1 [30]. The *pep1* gene was found to be specifically expressed during pathogenic development of *U. maydis* [30]. Pep1 deletion mutants form normal penetration structures but plant infection is stopped immediately upon epidermal penetration and this coincides with the elicitation of strong defense reactions in the host plant. Pep1 was found to localize to the plant apoplast where it particularly accumulates at sites of cell-to-cell passages of biotrophic *U. maydis* hyphae [30]. In addition, it became evident that Pep1 is not only essential for virulence of *U. maydis* but also for the related barley smut fungus *Ustilago hordei*, indicating a conserved function of Pep1 in other fungal biotrophs besides *U. maydis* [30]. However, so far it remained unclear why this effector plays such a crucial role for *Ustilago* infections. In this study, we present the functional characterization of Pep1 and demonstrate that it acts as inhibitor of host peroxidases.

## Results

### The Δ*pep1* mutant induces a hypersensitive response

**Figure 1:**
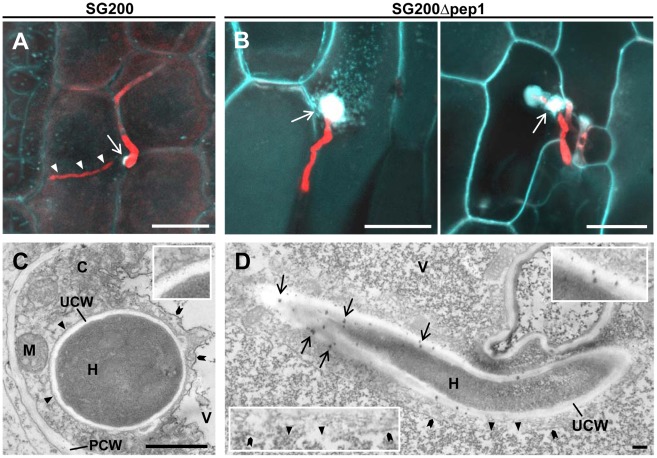
Characterization of plant defense responses to Δ*pep1* infections. (**A**) Appressorium (arrow) and penetrating hypha (arrow heads) of SG200RFP 24 hpi. Aniline blue staining indicates callose deposition at point of penetration which is suppressed upon establishment of biotrophy and intracellular growth. (**B**) Penetration attempts of SG200Δpep1RFP 24 hpi. Staining reveals formation of papillae below appressoria (arrows) and surrounding short invading hyphae. White bars: 20 µm. (**C**) TEM analysis of SG200 hypha in maize epidermis cell, stained with cerium chloride (CeCl_3_). The plant plasma membrane (arrow heads) surrounds the invading hypha (H). Magnification of the biotrophic interface shows no signs of CeCl_3_ staining (inset). V: plant vacuole, C: plant cytosol, UCW: *Ustilago maydis* cell wall, PCW: plant cell wall, M: mitochondrium, chevrons: tonoplast. (**D**) TEM picture of SG200Δpep1 invasion hypha (H) in maize epidermis cell. CeCl_3_ staining localizes at the biotrophic interface (arrows), revealing accumulation of ROS (magnification, upper right). Tonoplast (chevrons) is ruptured (arrow heads), indicating an induced hypersensitive response of the penetrated cell (inset, lower left). Black bars: 1 µm.

In a previous study we observed various plant responses such as autofluorescent cell wall depositions being induced by the *U. maydis pep1* deletion mutant(SG200Δpep1) [30]. To get additional insight in Pep1 function the defense activation, which is elicited by the *pep1* deletion mutant has now been studied in more detail. Maize leaves infected with SG200Δpep1 were stained with aniline blue, which selectively marks callose (1-3-β-D-glucose) that is synthesized as a defense response triggered by PAMP-induced elicitation [31]–[33]. Infection sites of the virulent *U. maydis* strain SG200RFP, which expresses cytoplasmic RFP [30], revealed only marginal callose accumulations surrounding the penetrating hypha (Figure 1A, S1A). In contrast, large depositions of callose were observed at points of attempted penetration by the RFP-expressing Δ*pep1* mutant strain SG200Δpep1RFP [30] (Figure 1B). Additionally, we performed cerium chloride (CeCl_3_) staining that allows the visualization of hydrogen peroxide (H_2_O_2_) by transmission electron microscopy (TEM) studies [34], [35]. TEM of maize epidermis cells infected by *U. maydis* strain SG200 revealed no ROS accumulation in these cells (Figure S1A). Intracellular hyphae were surrounded by an intact plant plasma membrane, reflecting the established biotrophic interface (Figure 1C). In contrast, around hyphae of SG200Δpep1 that penetrated epidermal cells we observed CeCl_3_ accumulations, indicating formation of ROS (Figure 1D). The H_2_O_2_ signal found in SG200Δpep1 infections particularly localized to the plant cell wall at sites of penetration (Figure S1B). CeCl_3_ staining also circumcised the penetrated cells, suggesting an ongoing hypersensitive response (HR) in these cells (Figure S2). The penetrated cells exhibited additional signs of HR such as ruptures of the tonoplast membrane and internal disintegration [36], (Figure 1D, S2).

**Figure 2:**
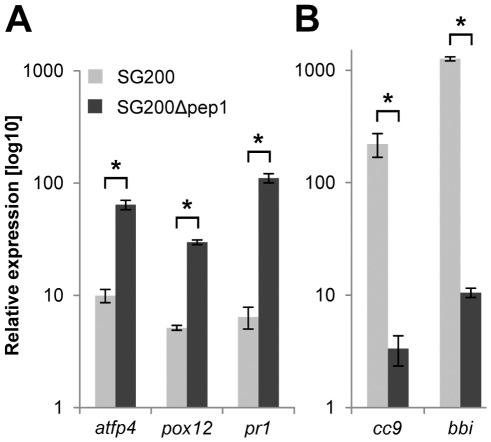
Regulation of JA and SA associated maize marker genes in response to *U. maydis* wild type versus SG200Δpep1 infections. Expression levels of SA/JA marker genes were determined by quantitative real-time PCR. The expression values represent three biological replicates and are shown relative to *GAPDH* expression in each sample. Leaf samples of mock, SG200 or SG200Δpep1 infected plants were taken after 2 dpi. Expression levels in mock infected plants were set to 1 and relative expression of marker genes was calculated for SG200 (light grey bars) and SG200Δpep1 (dark grey bars) samples. (**A**) Expression of SA marker genes *atfp4*, *pox12* and *pr1* 24 hours after infection with strain SG200Δpep1 or SG200, respectively. (**B**) Expression of JA marker genes *cc9* and *bbi* after infection with strain SG200Δpep1 or SG200, respectively. Data represent three biological replicates. P values have been calculated by an unpaired *t* test. Error bars show SEM. * P≤0.05.

Our previous microarray data on SG200Δpep1 infected maize leaves showed a strong induction of defense-associated host genes [30]. To further differentiate the transcriptional responses of maize to *U. maydis* wild type versus infections by the *pep1* deletion mutant, expression of typical JA-associated marker genes and SA-responsive transcripts was determined by qRT-PCR. As JA markers we used a Bowman Birk trypsin inhibitor [4] and the maize Cystatin-9 [37]. Both genes were strongly induced after SG200 infection, while their expression was only weakly induced in SG200Δpep1 infected maize leaves (Figure 2B). In contrast, the SA marker PR1 [5] as well as the SA-induced metal-ion binding protein ATFP4 [38]–[40] were upregulated specifically after SG200Δpep1 infection. Most interestingly, a gene encoding the maize peroxidase-12 (POX12) was highly induced upon SG200Δpep1 infections compared to wild type infections (Figure 2A). POX12 belongs to the class III peroxidases (NCBI: cd00693) of the plant heme-dependent peroxidase superfamily (Figure S3). Peroxidases of this class have also been reported to be involved in plant responses to pathogen attack and were involved in ROS production during the initial phase of the oxidative burst [8], [41], [42]. Summarizing our previous data with the present cytological and molecular observations we conclude that the Δ*pep1* mutant induces an oxidative burst response, eventually leading to plant cell death.

### Pep1 inhibits the oxidative burst

In light of the cell death-induction by the *pep1* deletion mutant, the major challenge was to elucidate how Pep1 interferes with the maize immune system. To functionally characterize Pep1, the open reading frames of *pep1* and *gfp*, which was used as a recombinant control protein, were fused to an N-terminal 6×His-tag and expressed in *E. coli* (see methods for details).

**Figure 3:**
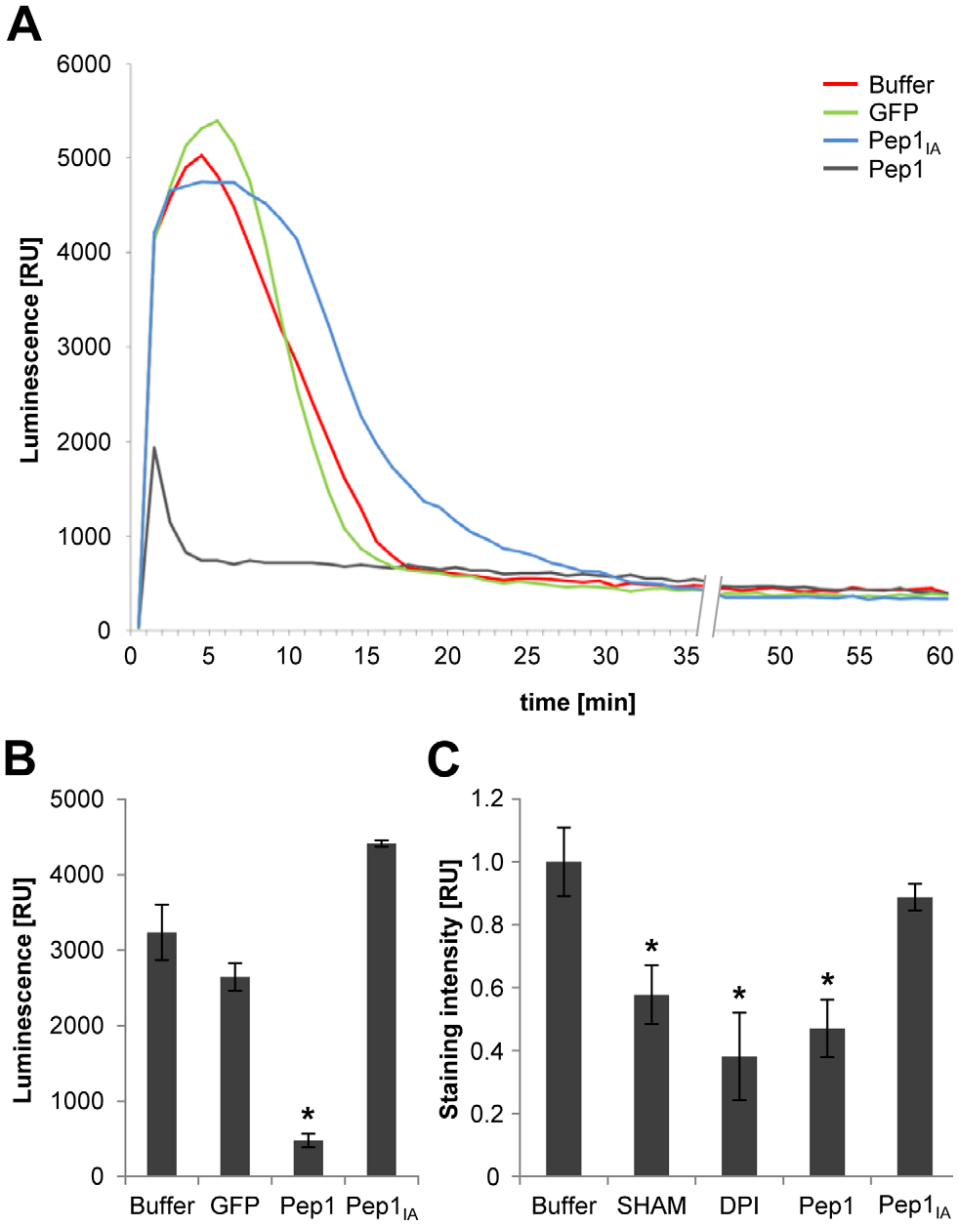
Inhibition of the elicitor triggered oxidative burst in maize leaves. (**A**) Luminol based readout to determine H_2_O_2_ production in maize leaf discs. The oxidative burst was elicited by the addition of chitosan (2.5 mg/ml) one minute after starting the measurement. Concentrations of recombinant Pep1, Pep1_IA_ and GFP proteins: 10 µM. (**B**) Quantification of elicitor triggered H_2_O_2_ production in maize leaf discs. The bars represent the integrated signal intensity of the average of 6 independent samples over the first 5 min after elicitation. (**C**) Quantification of chitosan induced H_2_O_2_ production in maize leaf discs based on xylenol orange staining. The peroxidase inhibitor SHAM (2 mM), NADPH-oxidase inhibitor DPI (5 µM) as well recombinant Pep1 protein (10 µM) cause a significant reduction of H_2_O_2_. Heat inactivated Pep1 (Pep1_IA_) does not influence the elicitor triggered oxidative burst. Data represent three biological replicates. P values have been calculated by an unpaired *t* test. Error bars show SEM. * P≤0.05.

As a test for the capability of Pep1 to suppress early plant defense responses, an oxidative burst was induced in maize leaf disks by the fungal elicitor chitosan (Figure 3A), or by heat inactivated *U. maydis* cells, respectively (Figure S4). In each case, the generation of ROS was visualized using a luminol-based readout. While the chitosan induced oxidative burst appeared transiently within 15 minutes after elicitation (Figure 3A), treatment with heat inactivated *U. maydis* caused a continuous burst that did not decrease within a measuring period of 60 minutes (Figure S4). Strikingly, recombinant Pep1 almost completely blocked the chitosan-induced oxidative burst (Figure 3A,B). Similarly, ROS production induced by heat inactivated *U. maydis* cells was blocked by Pep1 (Figure S4). In contrast, neither *E. coli* expressed GFP nor heat inactivated Pep1 protein inhibited the oxidative burst (Figure 3B, S4). These results show that Pep1 acts as an inhibitor of the elicitor-triggered oxidative burst, suggesting that Pep1 interferes with an essential component of this early defense reaction.

Next, we tested the origin of the PAMP triggered oxidative burst in maize leaves. To this end, maize leaf discs were treated with chitosan and the resulting H_2_O_2_ production was visualized *in vivo* by xylenol orange staining [43] (Figure 3C). Potential sources of ROS production under these experimental conditions were tested using either salicylhydroxamic acid (SHAM), an inhibitor of peroxidases [44], [45], or the NADPH-oxidase-inhibitor diphenylene iodonium chloride (DPI) [46], [47]. ROS production could be inhibited by both DPI and SHAM to similar extents (Figure 3C), suggesting that both NADPH-oxidase and peroxidase activity contributed to the PAMP-triggered oxidative burst in maize. While Pep1 did not interfere with the H_2_O_2_-induced color-change of xylenol orange (Figure S5), this assay showed an inhibition of the oxidative burst by Pep1 to similar levels as caused by treatment with either SHAM or DPI, respectively (Figure 3C).

### Scavenging of ROS enables the *Δpep1* mutant to penetrate the plant

**Figure 4:**
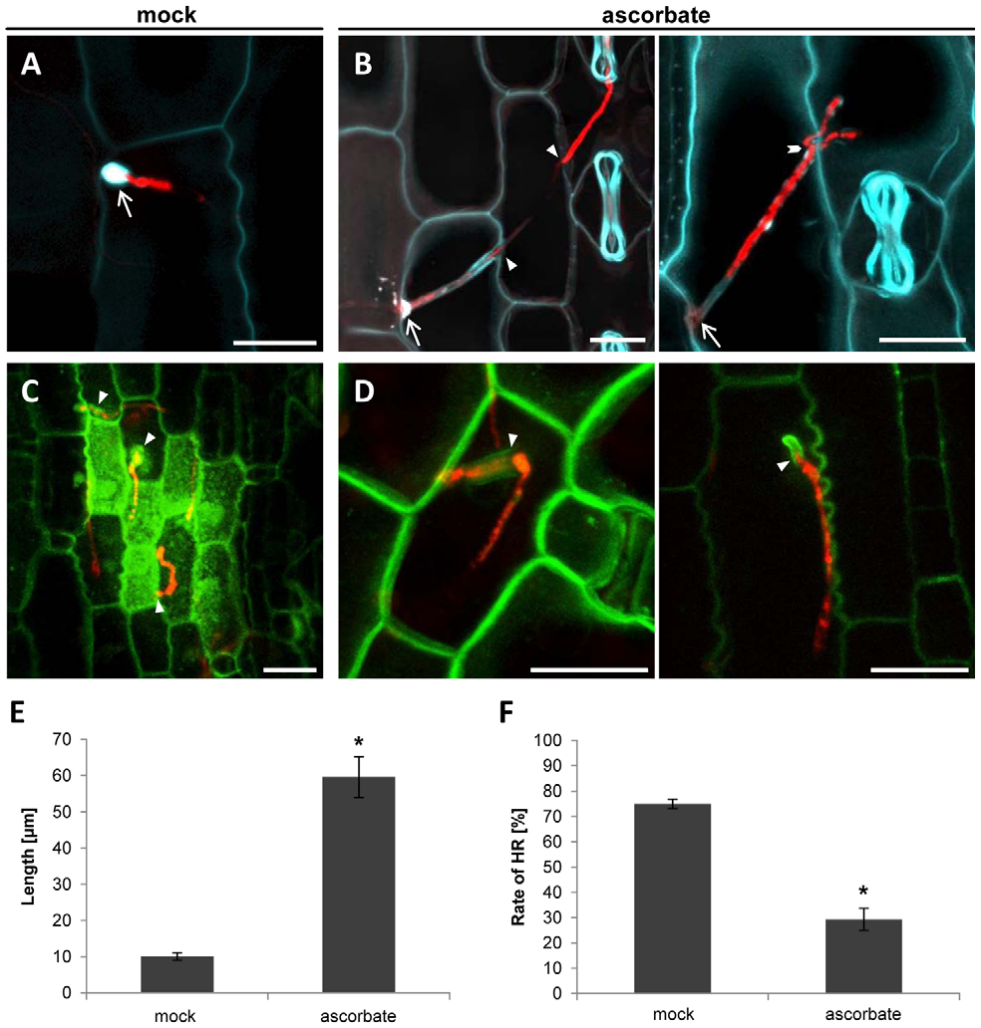
Scavenging of reactive oxygen species suppresses maize penetration resistance to the *Δpep1* mutant. (**A**) Aniline blue staining of SG200Δpep1RFP attempting to penetrate maize epidermis cell, 24 hpi. A papilla is formed at the point of penetration (arrow). (**B**) SG200Δpep1RFP penetrating the maize epidermis of plants treated with 5 µM ascorbate. Arrows mark penetration sites. The invading hyphae succeed in cell to cell penetrations (arrow heads); in some cases, proliferation of the hypha could be observed (chevron). (**C**) SG200Δpep1RFP on maize leaves expressing PIN1-YFP. Enhanced autofluorescence of penetrated cells as well as cells surrounding the penetration event indicate a HR reaction of the plant. (**D**) SG200Δpep1RFP is able to penetrate PIN1-YFP expressing maize leaves after the treatment with 5 µM ascorbate without eliciting enhanced autofluorescence. Bars: 20 µm. (**E**) Quantification of the length of intracellularly growing hyphae of SG200Δpep1 in maize leaves treated with 5 µM ascorbate compared to mock treated leaves. The addition of ascorbate leads to an average 6-fold increase of hyphal length. (**F**) Quantification of maize epidermal cells expressing visual signs of HR per penetration attempt by SG200Δpep1. The ascorbate treated plants show a ∼45% decrease in HR symptoms compared to the mock treated plants. Data represent three biological replicates. P values have been calculated by an unpaired *t* test. Error bars show SEM. * P≤0.05.

The ability of Pep1 to suppress the oxidative burst response is in line with the phenotype of the Δ*pep1* mutant (Figure 1; [30]). To test whether this particular function of Pep1 is relevant for *U. maydis* infection, pathogenic development of SG200Δpep1 was tested under conditions where ROS were scavenged through application of ascorbate. To this end, 5 mM ascorbate was applied to maize seedlings at the sites of SG200Δpep1 infections 12 and 24 hours after fungal inoculation, respectively. Confocal microscopy revealed that this treatment resulted in a drastic decrease in callose depositions at penetration sites (Figure 4A,B). Most importantly, under these conditions SG200Δpep1 was able to enter the leaf tissue with intracellular hyphae reaching up to 100 µm of length without eliciting a visible defense response (Figure 4B). In rare cases, branching of intracellular hyphae was observed, indicating proliferation of biotrophic SG200Δpep1 hyphae (Figure 4B). Quantification of infectious hyphae revealed an average increase of 600% of intracellular hyphal length after ascorbate treatment, compared to mock-treated control plants (Figure 4E). In addition, the rate of penetrated epidermis cells undergoing cell death was tested using a maize line expressing a YFP-tagged version of the auxin transporter PIN1 as a plasma membrane marker [30]. It got evident that ROS-scavenging significantly reduced Δpep1-induced cell death compared to mock treated maize leaves. While 75% of SG200Δpep1 infected maize cells collapsed in the controls (Figure 4C), only about 30% of penetration events caused such a response when ascorbate had been applied (Figure 4D,F). Maize leaves infected with SG200Δpep1 exhibited clusters of dead cells surrounding the infection site, while the addition of ascorbate led to reduced symptoms and the tissue stayed mostly alive and showed signs of chlorosis (Figure S6).

### Pep1 inhibits peroxidase activity and interacts with maize peroxidase-12

**Figure 5:**
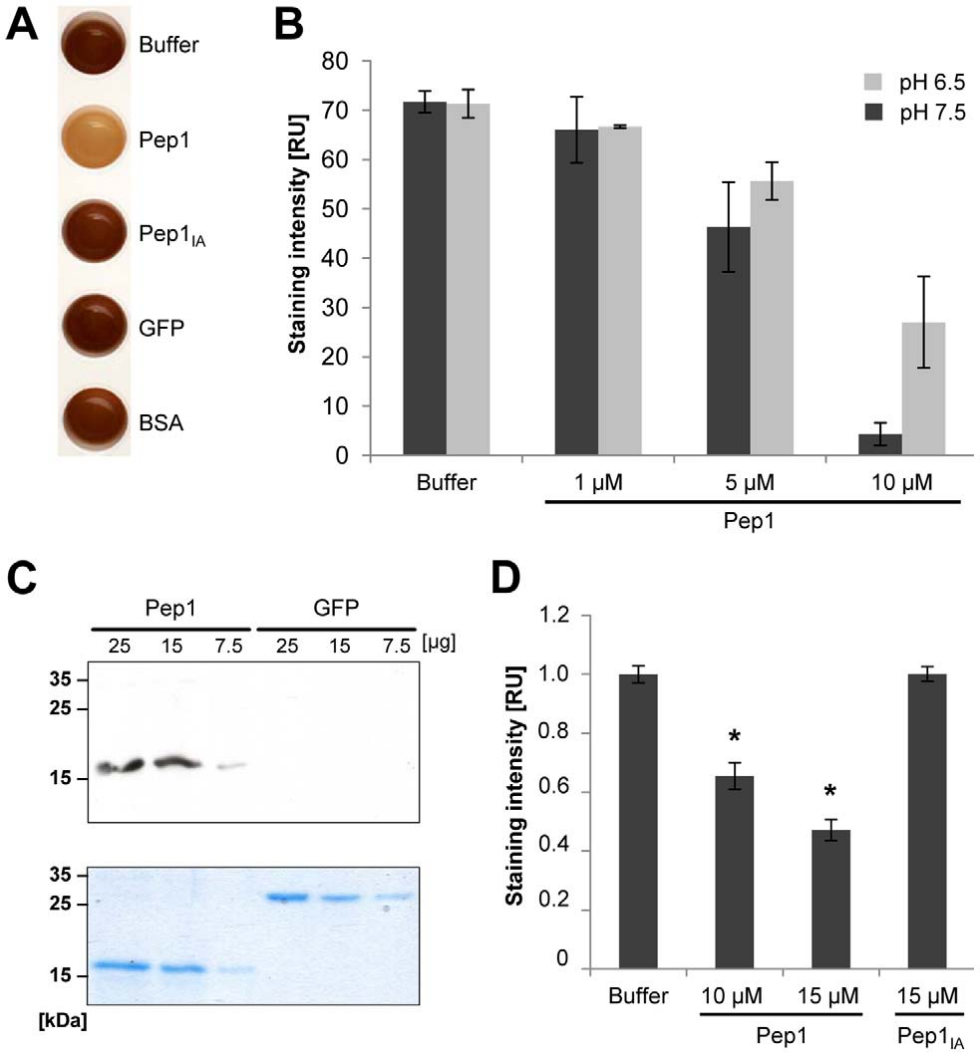
Pep1 directly inhibits peroxidase activity. (**A**) Measurement of HRP activity using an *in vitro* DAB assay. Dark coloration indicates peroxidase activity, visualized by the precipitation of DAB. The addition of purified GFP as well as heat inactivated Pep1 (Pep1_IA_) does not interfere with HRP activity. Addition of 5 µM native Pep1 results in reduced DAB precipitation, indicating suppressed HRP activity. (**B**) Quantification of DAB based HRP activity assay. Different concentrations of Pep1 were added to the assay solution at two different relevant pH values. Pep1 exhibits the ability of concentration dependent suppression of HRP activity at pH 6.5 and 7.5. (**C**) Far Western blot shows physical interaction of HRP with Pep1 (18.5 kDa) but not GFP (29.8 kDa) (upper panel). As a loading control, a separate gel was equally loaded and stained with coomassie blue (lower panel). (**D**) Peroxidase activity of maize apoplastic fluid was determined in a quantitative DAB assay. Recombinant native Pep1 and heat inactivated Pep1 (Pep1_IA_) were added to the reaction in respective concentrations. Data represent three biological replicates. P values have been calculated by an unpaired *t* test. Error bars show SEM. * P≤0.05.

The strong transcriptional induction of *pox12* in Δ*pep1* infections as well as the observed oxidative burst inhibition by Pep1 led us to consider the possibility that peroxidases could be a potential target of Pep1. To test this, a quantitative *in vitro* peroxidase assay was performed using commercial horseradish peroxidase (HRP) reacting with diaminobenzidine (DAB) in the presence of H_2_O_2_. In this assay, peroxidase activity of HRP results in the formation of a brown DAB precipitate. This reaction was also observed, when recombinant GFP or heat inactivated Pep1 were added to the assay (Figure 5A). In contrast, native Pep1 efficiently inhibited HRP activity (Figure 5A). This Pep1-driven peroxidase inhibition was found to depend on the Pep1 concentration, as well as on the pH (Figure 5B). To address the question whether Pep1 directly interacts with the peroxidase, a Far Western blot experiment was performed. Different amounts of Pep1 were blotted on a nitrocellulose membrane and incubated with HRP (Figure 5C). As a negative control, similar concentrations of recombinant GFP were blotted on the same membrane. Specific chemiluminescence signals showed that HRP was binding to Pep1 (Figure 5C). Furthermore, the intensity of chemiluminescence signals correlated with the amount of blotted Pep1 (Figure 5C), suggesting a direct interaction of Pep1 with HRP.

Given the finding that Pep1 resides in the plant apoplast [30], one could hypothesize that during *U. maydis* penetration, Pep1 might suppress ROS formation by inhibiting apoplastic peroxidase activity. To test the impact of Pep1 on maize peroxidases, apoplastic fluid of maize leaves was isolated. Extracted apoplastic fluids were tested for peroxidase activity using DAB precipitation. High levels of peroxidase activity were detected in the apoplastic fluid and this activity was inhibited specifically by native Pep1 in a concentration dependent manner (Figure 5D).

**Figure 6:**
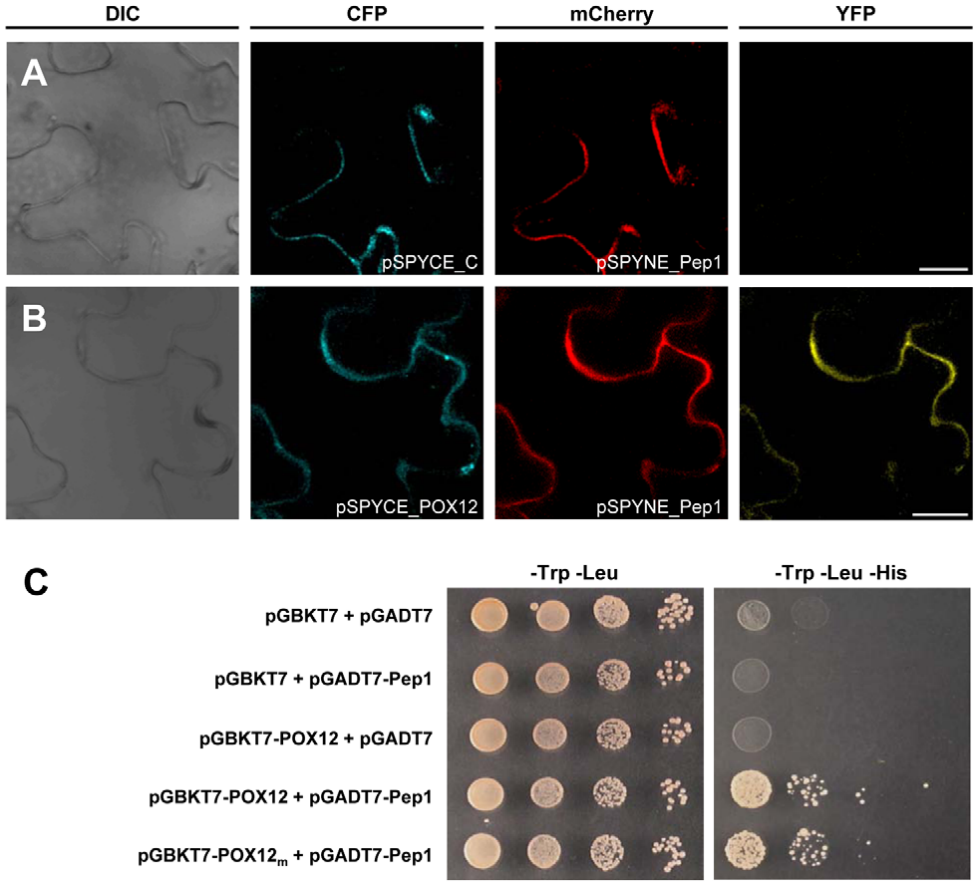
*In vivo* interaction of Pep1 with POX12. Confocal images in (A) and (B) show *N. benthamiana* epidermal cells expressing BiFC constructs. (**A**) A plant cell co-expressing pSPYCE_C and pSPYNE_Pep1. Blue and red channels show apoplastic co-localization of the respective signals. No complementation of fluorescence is observed in the YFP channel. (**B**) A cell co-expressing pSPYCE_POX12 and pSPYNE_Pep1. Both signals co-localize in the apoplast. The YFP channel exhibits YFP fluorescence with the same localization pattern indicating restoration of the YFP complex due to direct interaction of POX12 and Pep1. Bars: 25 µm. (**C**) Yeast-Two-Hybrid experiment confirming interaction of Pep1 and POX12. Mutation of the putative active site of POX12 (POX12_m_) did not abolish interaction with Pep1.

The finding that Pep1 inhibits HRP as well as apoplastic maize peroxidases suggested a rather unspecific interaction of Pep1 with peroxidases. The maize POX12, however, is not transcriptionally induced by H_2_O_2_ directly (Table S2), but displayed a particularly strong transcriptional activation upon Δ*pep1* infection (Figure 2) and no other maize peroxidase besides POX12 was transcriptionally induced upon SG200Δpep1 infection [30]. Furthermore, both HRP and POX12 belong to the type-III class heme-peroxidases, sharing 37% identity on the amino acid level and are highly conserved in the active domain (Figure S3). We therefore investigated whether Pep1 and POX12 physically interact inside the plant. To visualize the protein interactions *in vivo*, a modified split-YFP system was established, which allowed a microscopic localization of proteins also in cases where no fluorescence complementation took place. To this end, an mCherry tag was fused to the C-terminus of the N-terminal part of YFP (pSPYNE_R). Similarly, a CFP-tag was added to the C-terminal part of YFP (pSPYCE_C). Both constructs also contained an N-terminal secretion signal (for details see methods section) to facilitate apoplastic localization of the fusion proteins. Using *Agrobacterium tumefaciens* mediated transformation, the constructs were transiently expressed in *Nicotiana benthamiana* under the control of the 35S promoter. *N. benthamiana* cells expressing both pSPYCE_C and pSPYNE_R fused to *pep1* (pSPYNE_Pep1) showed apoplastic fluorescence signals for mCherry and CFP, indicating secretion of the fusion proteins (Figure 6A, S7A). However, expression of only the fluorescence fusion proteins with Pep1 did not result in any detectable YFP signal, demonstrating that no unspecific protein dimerization occurred (Figure 6A). Similarly, no YFP fluorescence was detected when pSPYNE_mCherry was co-expressed with pSPYCE_C fused to POX12 (pSPYCE_POX12) (Figure S7A). This shows that neither Pep1 nor POX12 caused any unspecific fluorescence. In contrast, cells that co-expressed pSPYNE_Pep1 and pSPYCE_POX12 showed a complementation of YFP fluorescence, which co-localized with the mCherry and CFP signals (Figure 6B). Similarly, co-expression of pSPYNE_POX12 (pSPYNE_R fused to POX12) and pSPYCE_Pep1 (pSPYCE_C fused to Pep1) resulted in YFP fluorescence complementation (Figure S7B). In addition, a yeast two hybrid experiment was performed to test interaction of Pep1 and POX12. Confirming the results obtained by fluorescence complementation, simultaneous expression of the two proteins in yeast restored growth on selection medium, indicating interaction of Pep1 and POX12 (Figure 6C). This interaction was also evident when the putative active site of POX12 [48] was mutagenized (see materials and methods for details), suggesting that Pep1 does not bind to the catalytic site of POX12 (Figure 6C). This specific fluorescence complementation by co-expression of Pep1 and POX12 fusion proteins confirms a direct physical interaction of Pep1 and POX12 *in vivo*, substantiating a biological function of Pep1 as a peroxidase inhibitor.

### POX12 activity is required for maize resistance to the *Δpep1* mutant

**Figure 7:**
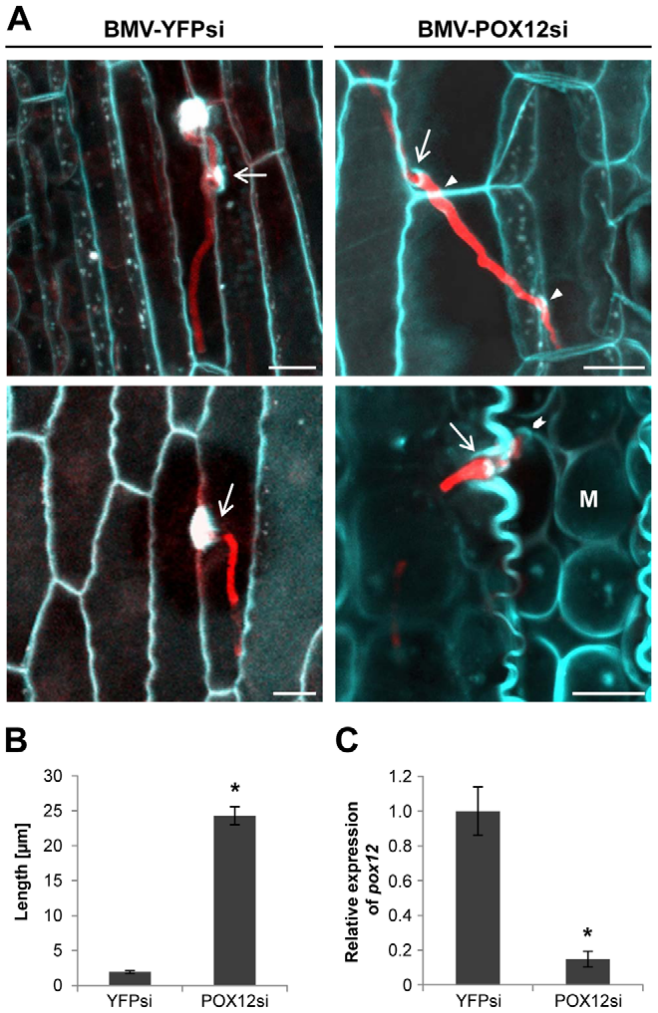
Silencing of *pox12* suppresses maize penetration resistance to the *Δpep1* mutant. (**A**) (left panel): Aniline blue staining of control plants (BMV-YFPsi) show formation of papillae at points of SG200Δpep1 penetration attempts. SG200Δpep1 is arrested directly upon penetration. (right panel): *pox12*-silenced (BMV-POX12si) maize plants infected with SG200Δpep1. Strain SG200Δpep1 successfully penetrates epidermal cells (arrows), shows cell to cell penetrations (arrow heads) and reaches the mesophyll layer (M, chevron) without eliciting visible plant defense responses. Bars: 10 µm (**B**) Quantification of intracellular hyphae length of *U. maydis* SG200Δpep1 on *pox12*-silencing plants compared to control plants. Silencing of *pox12* led to a significant, ∼10-fold increase in length of intracellular SG200Δpep1 hyphae. (**C**) *pox12* expression was quantified by quantitative real-time PCR using leaf samples of 8 independent *pox12*-silenced plants (BMV/POX12si) and 7 control plants (BMV/YFPsi) 48 h after *U. maydis* SG200Δpep1 infection (for details see methods section). Relative expression of *pox12* in BMV/YFPsi control plants was averaged and set to 1. Data represent three biological replicates. P values have been calculated by an unpaired *t* test. Error bars show SEM. * P≤0.05.

To test whether the Pep1-POX12 interaction is relevant for *U. maydis* interaction, the peroxidase gene was silenced in maize plants using a recently established virus-induced gene silencing (VIGS) assay that allows systemic gene silencing in maize during *U. maydis* interaction [49]. For POX12 silencing, two fragments of the coding region (see methods for details) were integrated into RNA3 of the *Brome mosaic virus* (BMV). Maize seedlings were inoculated with the resulting construct BMV-POX12si for subsequent *U. maydis* infection. Control plants were inoculated with a BMV silencing construct for the non-plant gene *YFP* (yellow fluorescent protein; BMV-YPFsi), which does not influence the maize – *U. maydis* interaction [49]. BMV inoculated plants were infected with SG200Δpep1 and fungal infection was monitored by confocal microscopy 48 hours after fungal infection. Similar to non-treated maize leaves (Figure 1B), BMV-YFPsi plants formed large papillae at sites of SG200Δpep1 infection and the mutant hyphae were stopped during epidermal penetration (Figure 7A). In contrast, silencing of POX12 led to a substantial increase in penetration efficiency as well as a reduction of visible plant defense responses (Figure 7A–C). In the POX12 silenced plants, biotrophic SG200Δpep1 hyphae were found to pass from cell to cell and they even colonized mesophyll cells (Figure 7A), a phenotype that has never been observed in control plants infected with SG200Δpep1. qPCR analysis confirmed an average reduction of POX12 transcript levels of 85% in the BMV-POX12si inoculated plants compared to the BMV-YFPsi controls (Figure 7C). From these results we conclude that POX12 activity contributes to maize resistance to the *Δpep1* mutant and that inhibition of this peroxidase by Pep1 is crucial for *U. maydis* infection.

## Discussion

Within the last years it became evident that secreted effector proteins are crucial for virulence of microbial plant pathogens [13], [50]. While the most detailed studies have been done on bacterial proteins [51], [52], also an increasing number of fungal effectors that contribute to virulence has been identified [53]–[57]. A characteristic feature of microbial effectors is the suppression of host immune responses [21], [25], [52], [58]. However, only little is known about the actual mode of action these proteins hold to interfere with the host immune system. Recently, a secreted *U. maydis* chorismate mutase has been shown to re-channel the host chorismate metabolism to avoid the synthesis of SA in order to suppress host defense [59].

At present Pep1 is the only known effector of *U. maydis* which is essential for establishment of compatibility. The *Δpep1* mutant is stopped by plant immune responses immediately upon initial penetration of the epidermis, suggesting that Pep1 interferes with a component of the first layer of defense. Here, we identified the secreted peroxidase POX12 as a direct Pep1 interaction partner. Silencing of POX12 led to a partial complementation of the *Δpep1* mutant phenotype suggesting that this peroxidase is responsible for arresting pathogenic development of the *Δpep1* mutant. These findings show that a fungal effector can directly interfere with the ROS-generating system of the host plant.

### The role of Pep1 in the suppression of the oxidative burst in maize

The *U. maydis Δpep1* mutant induces cell death of the infected epidermal cells. This coincides with H_2_O_2_ formation, which particularly accumulates in the apoplastic space and around the penetrating hyphae. Therefore, the ability of Pep1 to suppress the oxidative burst is perfectly in line with the mutant phenotype. The inability of the *Δpep1* mutant to establish biotrophy is reflected by the lacking induction of JA-responsive genes, which are markers for a compatible biotrophic interaction [4], [7]. In contrast, the *Δpep1* mutant strongly induced the expression SA-marker genes, which does not occur in *U. maydis* wild type infections [4]. This does also include the POX12, as defense-associated peroxidases typically are induced by SA [60]. An important finding for understanding the Pep1 function was that scavenging of ROS by an antioxidant suppresses callose deposition and cell death response upon epidermal penetration of the *Δpep1* mutant. Although ROS-scavenging might be considered as a rather unspecific intervention, its effect on *Δpep1*-induced plant responses suggests that suppression of apoplastic oxygen stress is sufficient to complement absence of Pep1 during epidermal penetration.

Main sources of ROS formation in the plant apoplast are the plasma membrane bound NADPH-oxidases [61], [62] and cell wall- or membrane-bound apoplastic peroxidases [11], [17], [63]. A couple of elegant studies, mainly done on the model plant *Arabidopsis*, demonstrated an important impact of NADPH-oxidase activity in ROS formation, cell death induction and thereby host immunity to microbial infection [13], [64]–[66]. However, there is evidence that depending on the host-pathogen interaction, different oxidative burst profiles relying on the activation of the NADPH-oxidase and/or the peroxidase systems are established [67], [68]. In barley, silencing of the NADPH-oxidase *HvRBOHF2* increased susceptibility to the barley powdery mildew *Blumeria graminis* f. sp. *hordei* as well as a reduction in wound induced cell death [69]. Interestingly, this phenotype was not associated with altered H_2_O_2_ production. The *HvRBOHF2* silencing barley plants showed normal H_2_O_2_ accumulation at penetration sites, papilla formation and hypersensitive reacting cells. In addition, the mutant plants showed a fully developed oxidative burst response after PAMP-treatment [69]. These findings indicate that in barley either functional redundancy among NADPH-oxidases, or action of additional components, i.e. peroxidases are involved in basal defense responses including the PAMP-triggered oxidative burst. Apoplastic peroxidases were found to be crucial during incompatible plant interactions [11] and their direct secretion was proposed to the sites of attempted pathogen invasion [63]. In wheat, the overexpression of a secreted class III peroxidase increased resistance to powdery mildew infection by potentiating the epidermal cell death response [70], which supports the role of defense-related peroxidases in plant defense to biotrophic pathogens.

### Interaction of Pep1 and plant peroxidases

Using *in vitro* assays, a physical interaction of Pep1 and HRP became evident, which correlated with a concentration-dependent inactivation of peroxidase activity by Pep1. In addition, Pep1 largely suppressed maize apoplastic peroxidase activity. To demonstrate interaction *in vivo* we selected the maize POX12 because of its strong transcriptional activation upon infection by the *pep1* deletion mutant, which was not found for any other peroxidase genes [30]. Our improved split-YFP method showed that apoplastic co-localization of either Pep1 or POX12 fusion proteins with only the fluorescence markers did not result in fluorescence signals, demonstrating specificity of the assay. Direct interaction with POX12 *in planta* verified the *in vitro* data of Pep1 physically binding to peroxidase. Regarding the Far Western approach it should be mentioned that Pep1-bound HRP apparently was not completely inactivated, because the assay is based on POX activity. This may result from the highly sensitive detection system that will visualize even marginal activity. However, together with the finding that Pep1 binds to POX12 as well as to HRP it indicates a rather unspecific interaction of Pep1 to peroxidases. This is also in line with the partial inhibition of the maize apoplastic peroxidase activity. The maize genome encodes for about 150 peroxidases [71],which makes it very likely that this apoplastic activity results from a mixture of different peroxidases that are sensitive to Pep1. However, a part of the apoplastic activity was not inhibited by Pep1, which might indicate that not all H_2_O_2_ producing enzymes present in the maize apoplast are targets of Pep1.

Despite the presence of multiple apoplastic peroxidases in maize, the POX12 appeared to be relevant for *U. maydis* infection. VIGS of POX12 lead to a significant rescue of the Δ*pep1* mutant phenotype. Particularly defense responses during epidermal penetration were suppressed in POX12 silencing maize leaves and *Δpep1* mutant hyphae were able to enter the mesophyll tissue. However, we did not observe the massive hyphal proliferation that is found in *U. maydis* wild type infections. Despite POX12 being the only maize peroxidase transcriptionally induced after Δ*pep1* infection, one must assume presence of other peroxidases that were not silenced in our approach. *In silico* prediction of siRNA formation (http://bioinfo2.noble.org/RNAiScan/RNAiScan.htm) deriving from the used POX12 silencing constructs revealed a potential co-silencing of only three closely related peroxidases (Genbank accessions: BT036551; BT036456; BT037744). However, none of the corresponding genes was induced in SG200Δpep1 infections [30]. Therefore, we consider it likely that remaining peroxidase activity in POX-silenced plants prevented a full complementation of the Δ*pep1* phenotype. Another possibility might be that Pep1 holds an additional function that adds to *U. maydis* virulence, which cannot be ruled out at present.

### Conclusions

Here we have shown that Pep1 has a crucial role for oxidative burst suppression via a direct inhibition of apoplastic peroxidases. This has uncovered an elementary virulence mechanism of *U. maydis*. In contrast to known effectors that interfere with specific plant signaling pathways, Pep1 suppresses PAMP-triggered immunity by scavenging one of its core components. The targeted peroxidases are a conserved, integral part of the first layer of plant defense responses. In line with this, Pep1 is also highly conserved in related pathogens such as the maize smut *Sporisorium reilianum* or the barley covered smut fungus *Ustilago hordei* [28], [30]. However, in published genome sequences of plant pathogens outside the group of *Ustilaginales*, no homologous proteins can be identified. Therefore it is an intriguing question whether other biotrophs developed analogous inhibitors of plant peroxidases, or if alternative mechanisms evolved to suppress basal plant defense.

## Materials and Methods

### Plant growth, culture media, fungal strains and infection conditions

For VIGS experiments, *Zea mays* L. cv Va35 [72] plants were grown in phytochambers at 28°C during the light period (26,000 lux; 14.5 h) and at 22°C during the dark period (9.5 h). For all other experiments, *Zea mays* cv. Early Golden Bantam was grown in a green house at 28°C during the light period (26,000 lux, 14.5 h) and 22°C during the dark period (9.5 h). *Nicotiana benthamiana* plants were grown at 22°C during the light period (26,000 lux; 14.5 h) and at 20°C during the dark period (9.5 h). For infections with *U. maydis*, a liquid culture of the strain SG200Δpep1 [30] was grown in YEPSL (0.4% yeast extract, 0.4% peptone and 2% sucrose) at 28°C shaking at 200 rounds min^-1^ (rpm) to an optical density (OD_600_) of 0.6–0.8. Cells were centrifuged at 900 g for 5 min, resuspended in H_2_O to an OD_600_ of 1.0 and used for infection of 17 day old maize seedlings (11 days after BMV inoculation).

To scavenge reactive oxygen species in SG200Δpep1 infections 5 mM ascorbate solution was applied to the infection site 12 and 24 h after *U. maydis* inoculation. Confocal observations were carried out 3 days after fungal infections. For staining of callose, aniline blue (Sigma, Taufkirchen, Germany) was used. Leaf samples were washed two times in 50% EtOH, followed by 2 washing steps in 100 mM Sodium-Phosphate buffer pH 9.0. Subsequently the samples were incubated in 0.05% (w/v) aniline blue in 100 mM Sodium-Phosphate buffer pH 9.0 for 1 h in the dark. Confocal microscopy of the samples was carried out in the staining solution. The *S. cerevisiae* strain AH109 (Clontech) was used for yeast two-hybrid interaction studies. Yeast cultures were grown in YPD full medium (1% yeast extract, 2% peptone, 1% glucose) or SD-Glucose minimal medium (0,67% yeast nitrogen base, 2% glucose) to an OD_600_ of 0.6–0.8 at 28°C shaking at 200 rpm. Yeast two-hybrid experiments were carried out following the Clontech Matchmaker GAL4 Two-Hybrid user manual. Selection of yeast transformants was conducted on SD-Glucose –Trp –Leu plates, selection for protein interaction was performed on SD-Glucose –Trp –Leu –His plates.

### Determination of reactive oxygen species in maize leaves

To quantify generation of reactive oxygen species from maize leaves in response to elicitor treatment, Luminol-based assays have been performed using maize leaf discs of 6 mm diameter. Luminol reaction solution was prepared as follows: 80 µM Luminol and 0.15 U/ml HRP (Sigma, Taufkirchen, Germany) were dissolved in 50 mM Sodium-Phosphate buffer pH 8.0. The assay volume was set to 1.5 ml, consisting of 1 ml Luminol reaction solution and 500 µl of elicitor solution. Luminescence measurements were carried out in a Tecan Infinite M200 plate reader (Tecan Group Ltd., Männedorf, Switzerland). Firstly a base line measurement was taken for 5 min before injection of the elicitor. After elicitation, measurements were continued for 180 min. Elicitor concentrations were 2 mg/ml glycol chitosan (f.c.) (Sigma) and 2×10^8^ cells/ml heat inactivated SG200 cells. For usage in the luminol assay, proteins were dissolved in 100 mM Sodium-Phosphate buffer pH 7.5+150 mM NaCl. For the colorimetric visualization of H_2_O_2_, each 12 leaf discs of 6 mm diameter were floated in 2 ml H_2_O. An oxidative burst was elicited by addition of 2.5 mg/ml chitosan to the assay. 5 min after elicitation, 120 µl of the water were harvested and H_2_O_2_ production was quantified with a xylenol orange based readout according to [17], [73]. Inhibitors/proteins were added 10 min prior to elicitation. Absorption measurements of the assay solution were undertaken in a Tecan Safire plate reader (Tecan Group Ltd., Männedorf, Switzerland). Background measurements were taken in the respective buffers and substracted from sample values.

### Peroxidase activity assays

*In vitro* HRP activity was visualized by DAB staining which was carried out in a clear 96 well micro titer plate (Greiner Bio-One, Frickenhausen, Germany). The assay solution consists of 2.7 mM DAB (Sigma, Taufkirchen, Germany), 0.375 U/ml HRP and 50 mM Sodium-Phosphate buffer (pH 6.5 or 7.5) in a total volume of 150 µl. DAB precipitation was initiated by the addition of 2 µl of 0.1% H_2_O_2_. If applicable, purified proteins were pre-incubated with the assay solution before the addition of H_2_O_2_. After 10 min the micro plate was scanned in an Epson V700 Photo flat bed scanner (Seiko Epson Inc., Tokyo, Japan) and staining intensity was quantified using Adobe Photoshop CS2 V. 9.02 (Adobe Systems Inc., San Jose, CA, USA) as follows. The image of the scanned plate was converted to gray scale and inverted subsequently. Now average grey values of each well were measured using the histogram tool. Blank wells filled with unstained buffer were measured as well and resulting grey values subtracted from sample values to eliminate the background.

For apoplastic fluid extraction, 100 Early Golden Bantam maize plants were grown under green house conditions at 28°C. After 7 days the seedling leaves were harvested and cut into pieces of 2–3 cm length, followed by evacuation under water in a vacuum chamber for 3×15 min at 400 mbar. The evacuated leaf sections were then stacked into packs of 20–30 and squeezed into the barrel of a 50 ml syringe so that the cut edges of the leaves faced the ends of the barrel. The barrel was then put into a 50 ml falcon tube with the needle hub facing downwards and spun for 20 min at 2000 g and 4°C. Afterwards the extracted apoplastic fluid was collected from the falcon tube and stored at −20°C. The POX activity of maize apoplastic fluid was visualized via *in vitro* peroxidase activity assay as described above, with the following modifications: The assay solution consists of 2.7 mM DAB (Sigma, Taufkirchen, Germany), 2 µl of 0.5 mg/ml maize apoplastic fluid and 50 mM Tris buffer (pH 7.5), in a total volume of 100 µl.

### Microscopy

Confocal images were taken on a TCS-SP5 confocal microscope (Leica, Bensheim, Germany), as described previously [30]. Fluorescence of YFP was elicited at 514 nm and detected at 520–540 nm, mCherry fluorescence was excited at 561 nm and detected at 590–630 nm, for detection of aniline blue (0.05% w/v in 0.1 M phosphate buffer pH 9.0) and cell wall autofluorescence, an excitation of 405 nm and detection at 435–480 nm were used.

Sample preparation for transmission electron microscopy (TEM) was performed by a modified protocol according to [34], [35]. Briefly, small pieces of leaves (1 mm^2^) were incubated for 1 h with a 5 mM cerium chloride (CeCl_3_) solution dissolved in 50 mM MOPS-buffer (3-(N-morpholino)propanesulfonic acid) at pH 6.5. Samples were then fixed in a mixture of 2.5% paraformaldehyde and 2.5% glutardialdehyde, dissolved in buffer at pH 7.2 for 90 min, rinsed in buffer (4 times, 10 min) and post fixed with 1% osmium tetroxide dissolved in buffer (pH 7.2). Dehydration was carried out in increasing concentrations of acetone (50%, 70%, 90% and 100%) for 2 times, 10 min for each step. The acetone was then exchanged with propylenoxide and the samples were then infiltrated with increasing concentrations of Agar 100 epoxy resin (30%, 60% and 100%). Samples were polymerized at 60°C for 48 h. Ultrathin sections (80 nm) were cut with a Reichert Ultracut S ultramicrotome and post stained for 5 min with a 2% lead citrate solution and for 15 min with 1% aqueous uranyl acetate before they were observed with a Philips CM10 TEM.

### Heterologous expression and purification of proteins

For expression of recombinant proteins in *E. coli*, strain Rosetta-gami(DE3)pLysS (Novagen/Merck, Darmstadt, Germany) was used. Expression vectors were based on pET15b (Novagen, Madison/USA). Cells were grown in dYT medium, containing 100 µg/ml ampicillin, 50 µg/ml kanamycin, 2.5 µg/ml tetracycline, 34 µg/ml chloramphenicol and 1% glucose, to the mid-logarithmic growth phase at 37°C. For protein overexpression cells were shifted to 28°C, followed by induction with 100 to 400 µM Isopropyl-β-D-thiogalactopyranosid (IPTG) (Sigma, Taufkirchen, Germany). After 4 hours cells were pelleted by centrifugation. Pellets were stored at −20°C.

Conditions for the purification of all the recombinant His-tagged proteins were optimized for maximal yield and purity by nickel affinity chromatography. The frozen cell pellet was resuspended in binding buffer (20 mM Tris, 500 mM NaCl, 20 mM imidazole, pH 7.9) supplemented with 500 µg/mL Lysozyme (Merck, Darmstadt, Germany), 0.1% Triton X-100 (Carl Roth, Karlsruhe, Germany) and incubated at room temperature for 20 min. The cells were disrupted by five times sonication at 4°C for 1 min with 1 min resting periods. The cellular debris was pelleted by centrifugation at 15,000 rpm and 4°C for 30 min. The purifications were carried out following the QIAexpressionist handbook (Qiagen, Hilden, Germany) with very little modification. The supernatant was applied to a gravity flow column, containing a bed volume of 1 ml Ni–NTA beads (Qiagen, Hilden, Germany), previously equilibrated with binding buffer. After incubation for 30 min at 4°C, the Ni–NTA column was washed with 5 bed volumes of binding buffer, followed by 5 bed volumes of washing buffer (20 mM Tris, 500 mM NaCl, 60 mM imidazole, pH 7.9). Recombinant proteins were then eluted from the Ni–NTA column with elution buffer (20 mM Tris, 500 mM NaCl, 500 mM imidazole, pH 7.9). For further assays the buffer was exchanged using illustra Nap25 columns (GE Healthcare, Buckinghamshire, United Kingdom). Depending on the subsequent assay, proteins were stored in Tris based storage buffer (100 mM Tris, 100 mM NaCl, 7.5), or in a sodium phosphate based storage buffer (100 mM sodium phosphate, 150 mM NaCl, pH 6.5 or 7.5). Proteins were concentrated using Amicon Ultra tubes (Milipore, Tullagreen, Ireland) with an exclusion size of 3 kDa. Finally 10% glycerol were added and proteins were stored at −20°C. The different stages of purification were monitored by SDS–PAGE. Protein concentrations were determined by Bradford assay employing BSA as a standard.

### Virus induced gene silencing of POX12

VIGS using BMV was performed as described previously [47]. To obtain BMV RNA1, RNA2 and RNA3, the plasmid pF1-11, pF2-2 and the different pB3-3 constructs were digested, individual transcripts were synthesized and the RNA integrities were tested as described previously [48].

To silence POX12, two siRNA-fragments were designed. POX12si-fragment-1 was corresponding to the bases 557–798 of the 1086 bp coding region of the *pox12* open reading frame. POX12si-fragment-2 was corresponding to the bases 766–937 of *pox12* open reading frame. Both fragments were individually integrated into two pB3-3 vectors as described [48]. To produce BMV containing POX12si-fragments, *Nicotiana benthamiana* plants were infected as described [70]. After inoculation, the leaves were harvested and ground in 0.1 M phosphate buffer, pH 6.0 (1∶10, w/v). The BMV titer was quantified by qPCR using primers specific for the minus strand of RNA1 (Table S1). All *N. benthamiana* extracts were adjusted by addition of 0.1 M phosphate buffer, pH 6.0, to the same virus titer of 2000 relative expression units compared with non-inoculated tobacco extracts, POX12si-fragmentswere mixed in a ratio of 1∶1 (v/v) and applied to the maize plants as described previously [47]. To determine the efficiency of *pox12* silencing, samples were taken two days after *U. maydis* infection of BMV inoculated plants and used for qRT-PCR as described previously [48]. Samples were cut diagonally to provide two samples for both qRT-PCR and microscopic analysis. Samples were shock frozen in liquid nitrogen and stored at −80°C. For RNA isolation, samples were ground to powder in liquid nitrogen and RNA was extracted with Trizol (Invitrogen, Karlsruhe, Germany) and purified using an RNeasy Kit (Qiagen, Hilden, Germany). After extraction, the First Strand cDNA Synthesis Kit (Fermentas, St. Leon-Rot, Germany) was used to reverse-transcribe 300 ng of total RNA with oligo(dT) primers for qRT-PCR. The qRT-PCR analysis was performed, using an iCycler machine (Bio-Rad, Munich, Germany) in combination with iQ SYBR Green Supermix (Bio-Rad). Cycling conditions were as follows: 2 min at 95°C, followed by 45 cycles of 30 sec at 95°C, 30 sec at 61°C and 30 sec at 72°C. Gene expression levels were calculated relative to *gapdh* as described in [49]. Error bars in all figures that show qRT-PCR data give the standard deviation that was calculated from the original CT (cycle threshold) values of three independent biological replicates.

### Far Western blot

Pep1 and GFP were overexpressed and purified as described above. Protein samples were prepared using 10 mM DTT and SDS loading buffer and boiled for 5 min. 25, 15 and 7.5 µg of total protein were separated by SDS-PAGE and transferred to a nitrocellulose membrane. After electroblotting, membranes were saturated with 5% non-fat dry milk in TBS-T (50 mM Tris-HCl, 150 mM NaCl, pH 7.6, 0.1% Tween-20 for 1 h at room temperature (RT). After blocking, the membrane was washed five times with TBS-T. Subsequently, the membrane was incubated over night at 4°C with horseradish peroxidase (HRP) (Sigma, Taufkirchen, Germany) dissolved in TBS-T at a concentration of 25 µM. Then, the membrane was washed five times with TBS-T and signals were detected by chemiluminscence detection using ECL Plus Western Blot detection reagent (GE Healthcare).

### Nucleic acid manipulations

Standard molecular biology methods were used according to [74]. All restriction enzymes used in this study were purchased from New England Biolabs (Frankfurt/Main, Germany).

For protein overexpression of Pep1 and GFP the *E. coli* expression vector pET15b (Novagen, Schwalbach, Germany) was used. *pep1* was amplified from *U. maydis* SG200 DNA, using Primers O19 and O20 (Table S1). *gfp* was amplified from the p123 vector [75]. PCR products were cloned into the pET15b vector via NdeI and BamHI restriction sites. Isolation of genomic *U. maydis* DNA was performed as described previously [76]. PCR was performed using Phusion High-Fidelity (NEB, Frankfurt/Main, Germany). The PCR products of the different genes were cleaned up before digestion, using the Wizard SV Gel and PCR Clean-Up System (Promega, Mannheim, Germany) and ligated into the pET15b expression vector. The vectors were transformed into DH5α cells (Invitrogen, Karlsruhe, Germany), and then plated on YT-agar plates containing 100 µg/ml ampicillin. At least three colonies were picked and grown over night in 2 ml dYT medium containing 100 µg/ml ampicillin. Plasmids were extracted using QIAprep system (Qiagen, Hilden, Germany) and cleaned by Wizard SV Gel and PCR Clean-Up System. After sequencing, one correct construct was transformed into *E. coli* Rosetta-gami(DE3)pLysS (Novagen/Merck, Darmstadt, Germany).

Constructs for the microscopic interaction studies via BiFC were based on pUC-SPYNE-35S and pUC-SPYCE-35S [77]. Plasmids were modified as follows. To obtain the additional fluorescence tag, genes encoding for CFP and mCherry respectively were amplified using primers O23-O25 (Table S1) adding a RSIATA spacer sequence. PCR products were cloned into the BiFC-vectors via XhoI and XmaI restriction sites. ORFs from the BiFC vectors were then digested with HindIII and EcoRI and ligated into pGreen0000 [78]. To remove excess restriction sites from the obtained vectors, two inverse PCR steps were added using primers O26-O29 (Table S1). PCR fragments were digested with NdeI and HindIII, followed by religation resulting in the vectors pGreen_SPYCE-CFP and pGreen_SPYNE-mCherry. Subsequently a codon optimized *pep1* gene that carries a secretion signal from the legumine B4 gene from *Vicia faba* (GenBank: X03677.1) was then amplified using primers O30 and O31 (Table S1) and inserted into pGreen_SPYNE-mCherry and pGreen_SPYCE-CFP via restriction sites BamHI and XhoI. The POX12 gene from *Z. mays* was amplified using primers O32 and O33 (Table S1) and inserted into pGreen_SPYCE-CFP via restriction sites BamHI and XhoI. Control vectors pSPYCE_C and pSPYNE_R were generated by removing the pep1 gene from pGreen_SPYNE-mCherry-Pep1 and pGreen_SPYCE-CFP-Pep1 via inverse PCR using primers O34 and O35 (Table S1) and religation, leaving the legumine B4 signal peptide from *V. faba* in the vector, resulting in secreted BiFC constructs as negative controls.

Created BiFC vectors were transformed into *A. tumefaciens* GV3101 cells by electroporation (1.5 kV). *Agrobacterium* mediated transient transformation of *N. benthamiana* was carried out following the protocol of [79]. For confocal microscopy, leaf discs with a diameter of 6 mm were cut from the infected leaf areas and immediately observed in the microscope.

Constructs for Yeast-Two-Hybrid interaction studies were based on the vectors pGBKT7 and pGADT7 (Clontech). The *pep1* gene was cloned into pGADT7 using the primer pair O36/O37 and the restriction sites XmaI and XhoI. The *pox12* gene was cloned into pGBKT7 using the primers O38/O39 and the restriction sites NdeI and EcoRI. The putative active site of POX12 [48] was mutagenised by exchanging Arg74, His78 and His207 to Alanine. Phe77 was exchanged by Valine. Respective point mutations were introduced to the *pox12* gene at positions using the primers O40 and O41 with the Quik Change Multi Site Directed Mutagenesis Kit (Agilent Technologies, Santa Clara, CA).

To generate the pB3-3/POX12si constructs, the primers O13-O16 (Table S1) were designed for two RNAi antisense fragments of POX12 (GenBank: EU964425.1). All primers contained a HindIII restriction site for integration into pB3-3 (Table S1). Cloning and integration into pB3-3 vector was done as described in [49]. The following maize gene fragments were inserted in antisense orientation in pB3-3 vector to RNA3: for POX12-fragment 1, 248 bp; and for POX12-fragment 2, 178 bp. As a silencing control pB3-3/YFPsi of our previous study was used [49]. For *in silico* analysis of siRNA formation and the silencing specificity of maize sequences, the software tool SIRNA SCAN (http://bioinfo2.noble.org/RNAiScan.htm) was used. Predictions were made using data from the J. Craig Venter Institute maize tgi v16 database. pF1-11 and pF2-2 were provided by X. S. Ding & R. S. Nelson [72]. For RNA isolation, infected leaf areas of each 30 maize seedlings were pooled, ground in liquid nitrogen, extracted with Trizol (Invitrogen, Karlsruhe, Germany) and purified using an RNeasy Kit (Qiagen, Hilden, Germany).

Expression of Zm-*pr1* (ZMU82200), Zm-*pox12* (ACG36543), Zm-*atfp4* (NP_001152411.1), Zm-*cc9* (BN000513.1) and Zm-*bbi* (EU955113.1) were analyzed by qRT-PCR using primers O1–O12 (Table S1). After extraction, the First Strand cDNA Synthesis Kit (Fermentas, St. Leon-Rot, Germany) was used to reverse-transcribe 1 µg of total RNA with oligo(dT) primers for qRT-PCR. The qRT-PCR analysis was performed as described above. Gene expression levels were calculated relative to *gapdh* expression levels as shown previously [49].

Sequence alignments of conserved domains among class III heme-peroxidases according to [48] was done using clone manager 9.1 software (Sci-Ed software, Cary, USA). For sequence assembly *pox12* (EU964425.1) served as reference sequence.

## Supporting Information

Figure S1 CeCl_3_ staining of penetration events shows ROS accumulation at SG200Δpep1 penetration sites.
(PDF)

Figure S2 SG200Δpep1 infected cells undergo HR.
(PDF)

Figure S3 Conserved domains among class III heme-peroxidases.
(PDF)

Figure S4 Pep1 inhibits maize oxidative burst triggered by heat inactivated *Ustilago maydis* cells.
(PDF)

Figure S5 Pep1 does not inhibit the xylenol orange assay.
(PDF)

Figure S6 Macroscopic phenotype of maize leaves three days after infection with strain SG200Δpep1.
(PDF)

Figure S7 *In vivo* visualization of a direct interaction of Pep1 with POX12.
(PDF)

Table S1 PCR primers used in this study.
(PDF)

Table S2 Relative expression of *pox12* in response to H_2_O_2_.
(PDF)
