# Supplementary material for: The Ustilago maydis Effector Pep1 Suppresses Plant Immunity by Inhibition of Host Peroxidase Activity
Source: PLoS Pathog. 2012 May 10;8(5):e1002684. doi: 10.1371/journal.ppat.1002684 (PMC3349748; doi:10.1371/journal.ppat.1002684)
Supplement: Figure S1 — CeCl3 staining of penetration events shows ROS accumulation at SG200Δpep1 penetration sites. (PDF) [file ppat.1002684.s001.pdf]

Figure S1

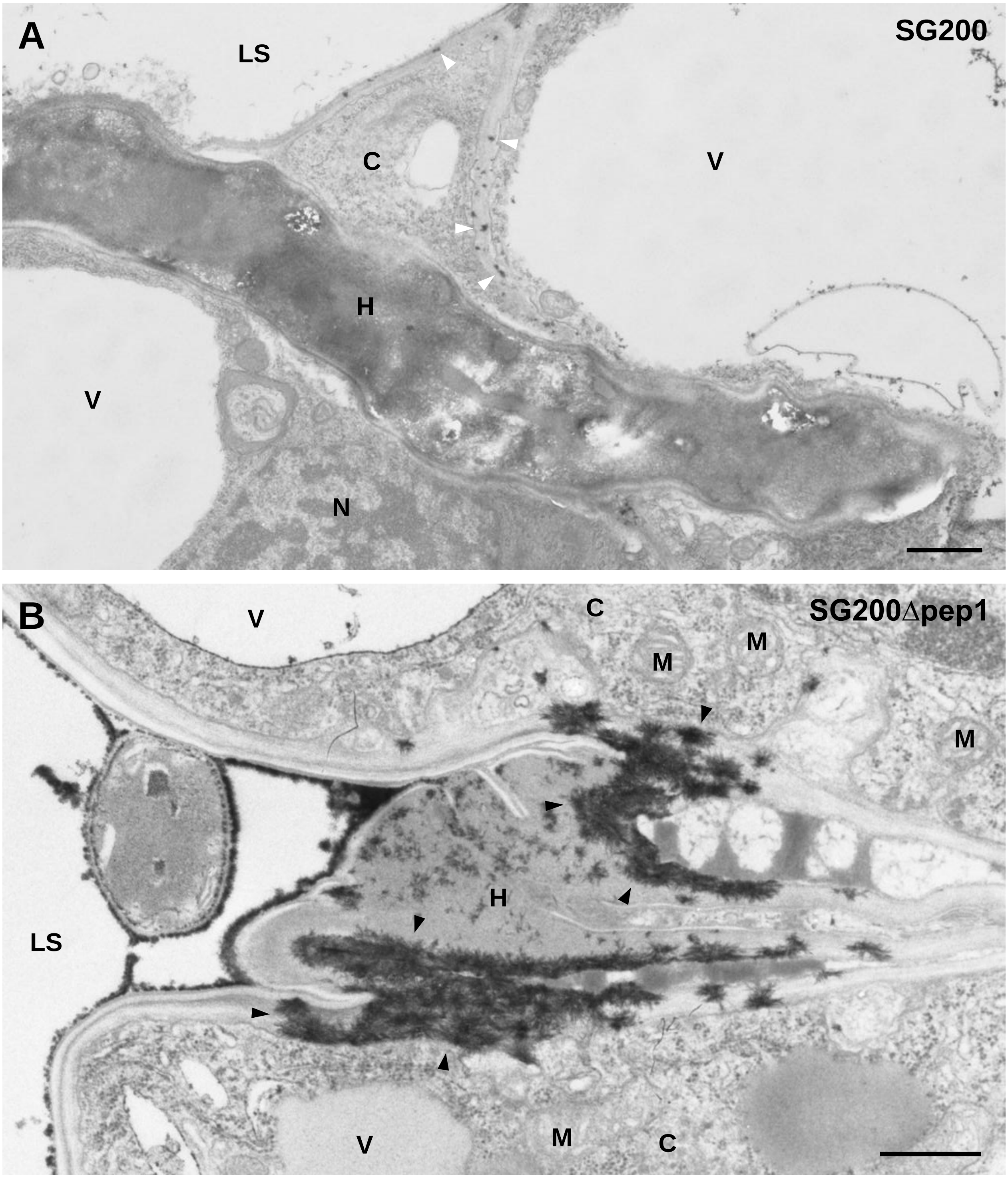

Supplementary Figure 1. $CeCl_3$ staining of penetration events shows ROS accumulation at SG200Δpep1 penetration sites.

(A) Penetration event of SG200 stained with $CeCl_3$. In some cases a signal could be observed in the plant cell wall distant from the invading hypha (white arrow heads). H: *Ustilago maydis* hypha V: plant vacuole, C: plant cytosol, N: nucleus, M: mitochondrium, LS: leaf surface. **(B)** Penetration event of SG200Δpep1 stained with $CeCl_3$. Dense signals surrounding the invading hypha (black arrow heads) indicate massive oxidative stress. Bars: 1 µm.
