# Supplementary material for: The Ustilago maydis Effector Pep1 Suppresses Plant Immunity by Inhibition of Host Peroxidase Activity
Source: PLoS Pathog. 2012 May 10;8(5):e1002684. doi: 10.1371/journal.ppat.1002684 (PMC3349748; doi:10.1371/journal.ppat.1002684)
Supplement: Figure S2 — SG200Δpep1 infected cells undergo HR. (PDF) [file ppat.1002684.s002.pdf]

Figure S2

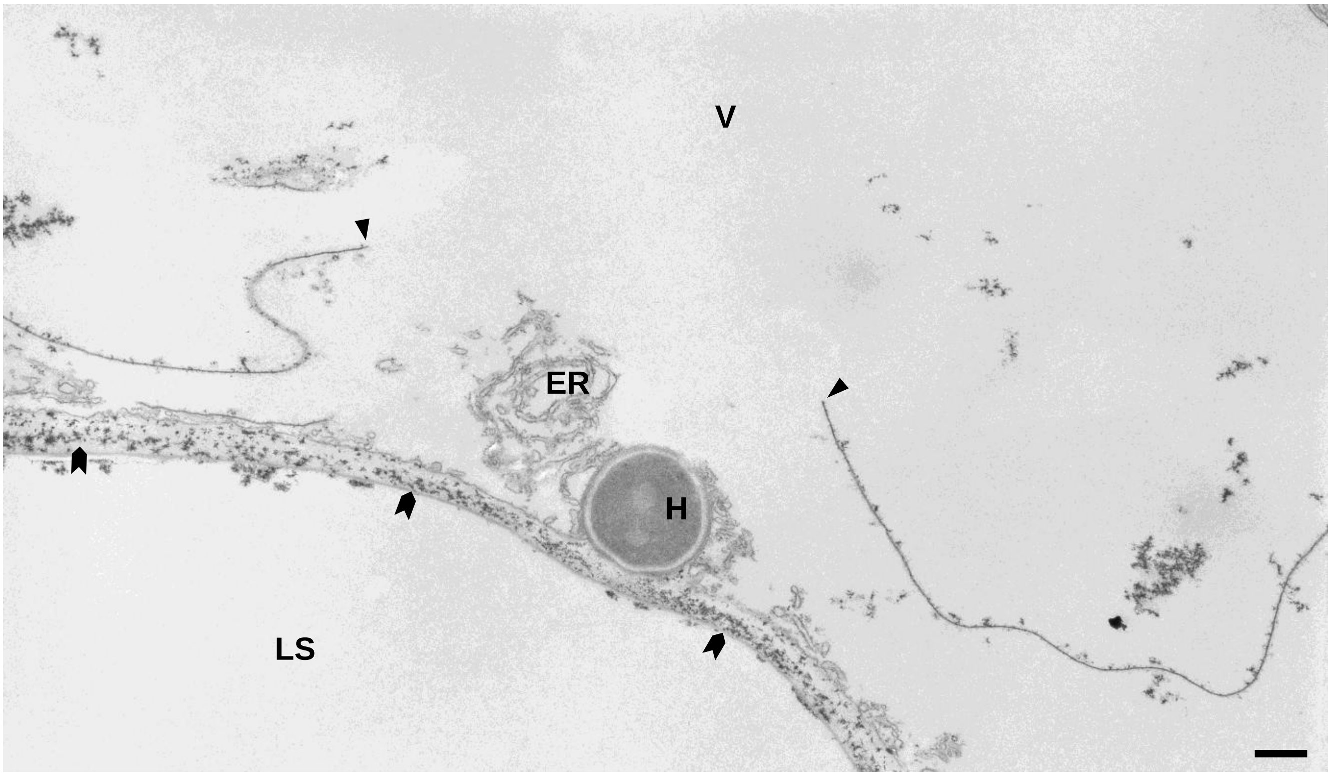

Supplementary Figure 2. SG200∆pep1 infected cells undergo HR.

TEM image of a maize epidermis cell undergoing HR after penetration by SG200∆pep1. Note the ruptured tonoplast (arrow heads) and severe oxidative stress indicated by $CeCl_3$ accumulation in the cell wall (chevrons). H: *Ustilago maydis* hypha, ER: endoplasmic reticulum, V: vacoule, LS: leaf surface. Bar: 1 µm.
