# Supplementary material for: The Ustilago maydis Effector Pep1 Suppresses Plant Immunity by Inhibition of Host Peroxidase Activity
Source: PLoS Pathog. 2012 May 10;8(5):e1002684. doi: 10.1371/journal.ppat.1002684 (PMC3349748; doi:10.1371/journal.ppat.1002684)
Supplement: Figure S3 — Conserved domains among class III heme-peroxidases. (PDF) [file ppat.1002684.s003.pdf]

Figure S3

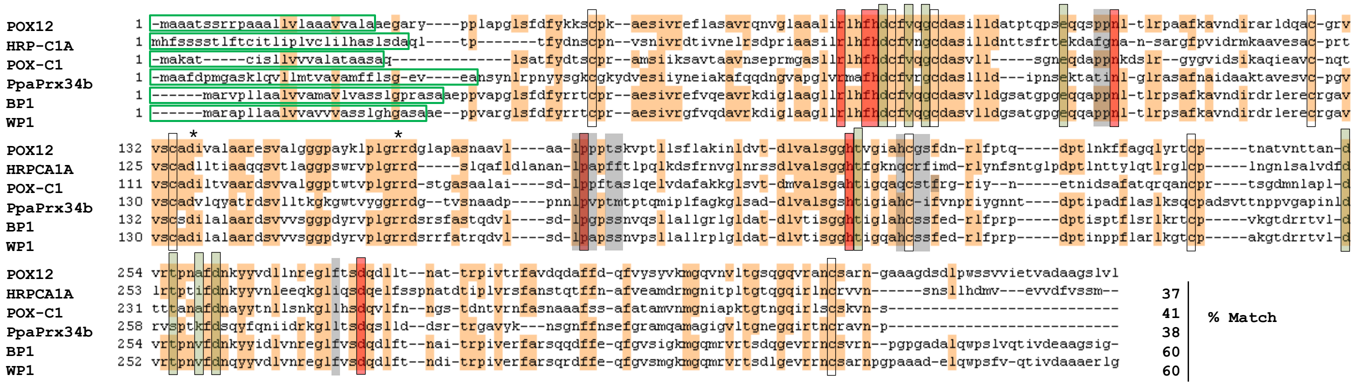

Supplementary Figure 3. Conserved domains among class III heme-peroxidases. Amino acid sequence alignment of POX12 with selected members of secreted class III heme-peroxidases according to [77]. Green boxes: Signal peptide region (according to signal peptide prediction by SMART (http://smart.embl-heidelberg.de)) Red shaded boxes: conserved residues important for catalytic activity. Gray shaded boxes: substrate access channel lining residues. Green shaded boxes: Ca^{2+}-binding residues. Black boxes: Cysteine residues forming disulfide bridges. Predicted Asp-Arg salt bridge residues are indicated by *. Peroxidase used for the alignment: POX12, (*Zea mays*) (ACG36543); HRP-C1A, (*Armorica rusticana*) (P00433.2); POX-C1, (*Oryza sativa*) (AAF65464.2); PpaPrx34b (*Physcomitrella patens*) (XP_001777299.1); BP1 (*Hordeum vulgare*) (Q40069); WP1 (*Triticum aestivum*) (AF525425).
