# Supplementary material for: The Ustilago maydis Effector Pep1 Suppresses Plant Immunity by Inhibition of Host Peroxidase Activity
Source: PLoS Pathog. 2012 May 10;8(5):e1002684. doi: 10.1371/journal.ppat.1002684 (PMC3349748; doi:10.1371/journal.ppat.1002684)
Supplement: Figure S4 — Pep1 inhibits maize oxidative burst triggered by heat inactivated Ustilago maydis cells. (PDF) [file ppat.1002684.s004.pdf]

Figure S4

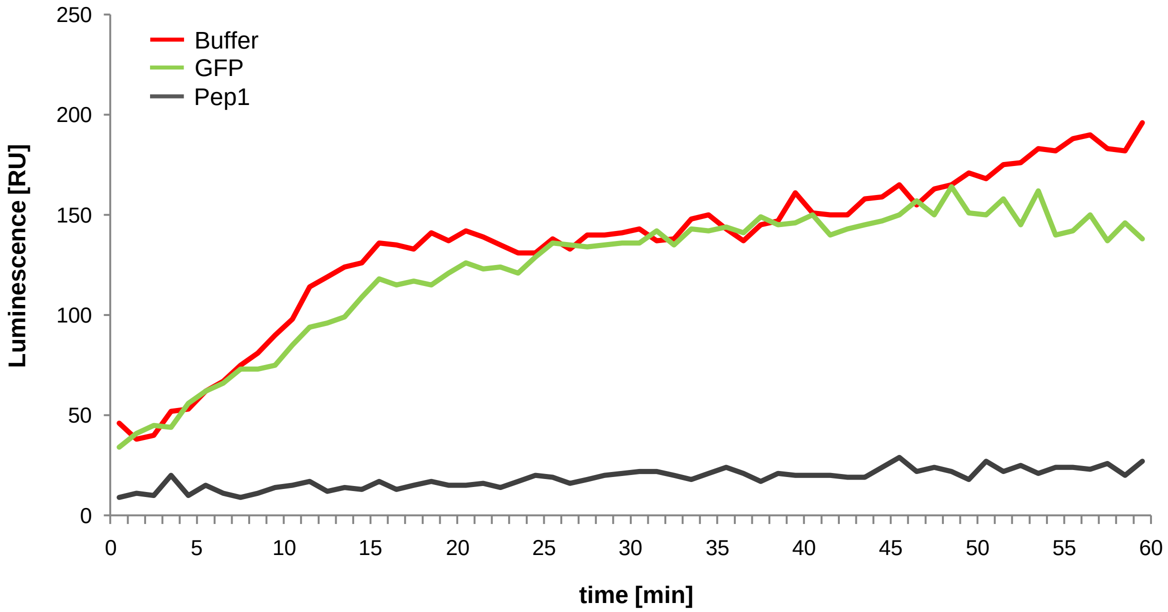

Supplementary Figure 4. Pep1 inhibits maize oxidative burst triggered by heat inactivated *Ustilago maydis* cells. Luminol based graphical representation of H_2O_2 production in maize leaf discs, elicited by the addition of $2x10^8$ c/ml heat inactivated cells of *Ustilago maydis* strain SG200 at time point 1 min. Concentrations of recombinant Pep1 and GFP proteins: 10 µM.
