# Supplementary material for: The Ustilago maydis Effector Pep1 Suppresses Plant Immunity by Inhibition of Host Peroxidase Activity
Source: PLoS Pathog. 2012 May 10;8(5):e1002684. doi: 10.1371/journal.ppat.1002684 (PMC3349748; doi:10.1371/journal.ppat.1002684)
Supplement: Figure S5 — Pep1 does not inhibit the xylenol orange assay. (PDF) [file ppat.1002684.s005.pdf]

Figure S5

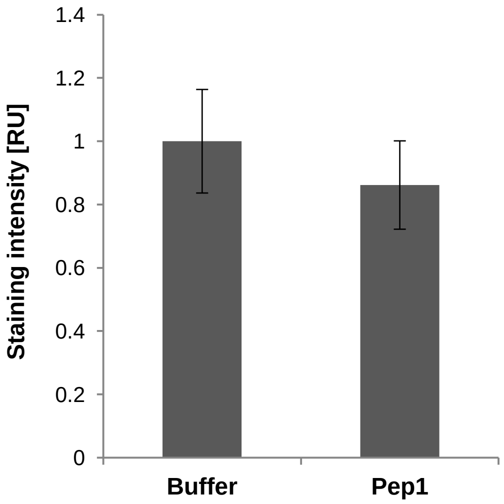

Supplementary Figure 5. Pep1 does not inhibit the xylenol orange assay. Xylenol Orange assay with 100 mM Tris Buffer pH 7.5, 150 mM NaCl and Pep1. Color reaction was triggered by the addition of 0.1% H_2O_2. There is no statistically significant difference between the buffer control and the Pep1 sample. Data represent three replicates. P values have been calculated by an unpaired *t* test. Error bars show SEM. P = 0.5229.
