# Supplementary material for: The Ustilago maydis Effector Pep1 Suppresses Plant Immunity by Inhibition of Host Peroxidase Activity
Source: PLoS Pathog. 2012 May 10;8(5):e1002684. doi: 10.1371/journal.ppat.1002684 (PMC3349748; doi:10.1371/journal.ppat.1002684)
Supplement: Figure S6 — Macroscopic phenotype of maize leaves three days after infection with strain SG200Δpep1. (PDF) [file ppat.1002684.s006.pdf]

Figure S6

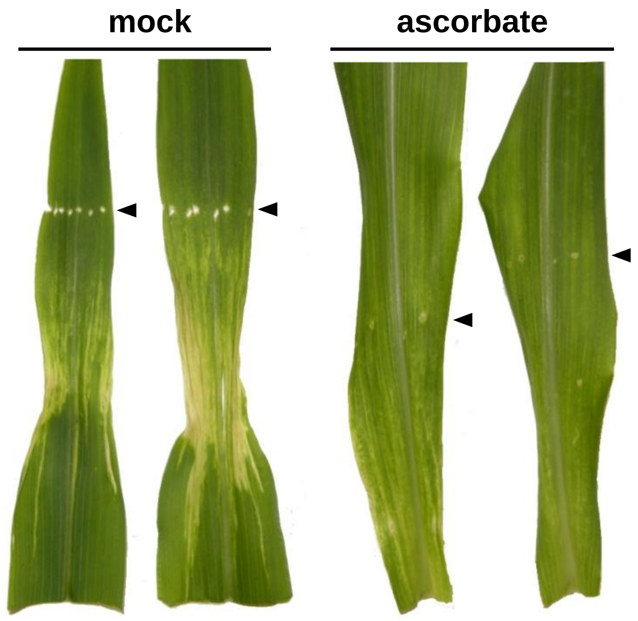

Supplementary Figure 6. Macroscopic phenotype of maize leaves three days after infection with strain SG200∆pep1.
Mock infected plants have been injected with water 12 and 24 hpi and exhibit necrotic areas below points of infection. Plants treated with 5 mM ascorbate 12 and 24 hpi show less signs of cell death and mostly chlorotic tissue below points of infection (arrow heads mark infection sites).
