# Supplementary material for: The Ustilago maydis Effector Pep1 Suppresses Plant Immunity by Inhibition of Host Peroxidase Activity
Source: PLoS Pathog. 2012 May 10;8(5):e1002684. doi: 10.1371/journal.ppat.1002684 (PMC3349748; doi:10.1371/journal.ppat.1002684)
Supplement: Figure S7 — In vivo visualization of a direct interaction of Pep1 with POX12. (PDF) [file ppat.1002684.s007.pdf]

Figure S7

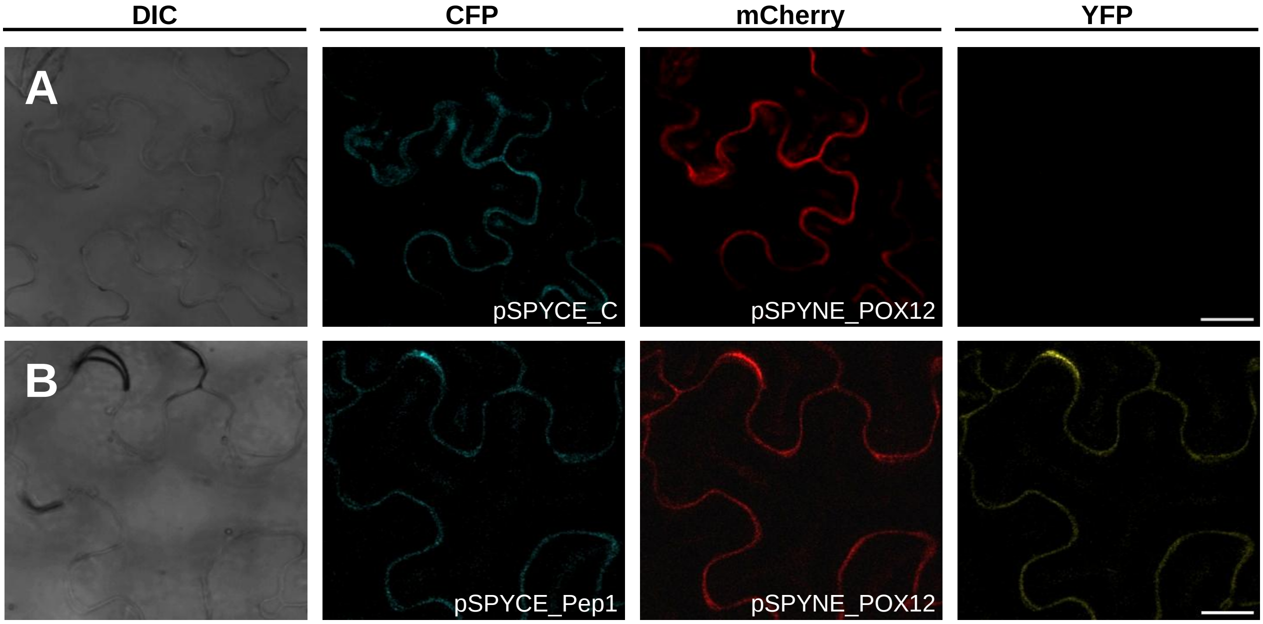

Supplementary Figure 7. *In vivo* visualization of a direct interaction of Pep1 with POX12.
A: A cell co-expressing pSPYCE_C and pSPYNE_POX12. Blue and red channels show apoplastic localization of the respective signals. No fluorescence complementation is observed in the YFP channel. **B:** A *N. benthamiana* cell co-expressing pSPYCE_Pep1 and pSPYNE_POX12. Both signals co-localize in the apoplast. The YFP channel exhibits YFP fluorescence with the same localization pattern indicating a restoration of the YFP complex due to direct interaction of POX12 and Pep1. Bars: 25 µm.
