# Supplementary material for: The Ustilago maydis Effector Pep1 Suppresses Plant Immunity by Inhibition of Host Peroxidase Activity
Source: PLoS Pathog. 2012 May 10;8(5):e1002684. doi: 10.1371/journal.ppat.1002684 (PMC3349748; doi:10.1371/journal.ppat.1002684)
Supplement: Table S1 — PCR primers used in this study. (PDF) [file ppat.1002684.s008.pdf]

Table S1 PCR primers used in this study

qRT Primers

#	Name	Reference	Sequence 5'→3'	Application
O1	CC9-qRT-fw	BN000513.1	TATGGGTCCTTGACGTTCTC	Quantification of *cc9* expression by q(RT)PCR
O2	CC9-qRT-rv	BN000513.1	GGATCATCCGTAGCCATCTG	
O3	BBI-qRT-fw	EU955113.1	CCGACATCCTCTTCAACTTCTG	Quantification of *bbi* expression by q(RT)PCR
O4	BBI-qRT-rv	EU955113.1	TTCTCTGAAGCGGCACAC	
O5	POX12-qRT-fw	ACG36543	CTGAACAAGTTCTTCGCGG	Quantification of *pox12* expression by q(RT)PCR
O6	POX12-qRT-rv	ACG36543	AGGTCCACGTAGTACTTGTTG	
O7	PR1-qRT-fw	ZMU82200	ACTACGTGGACCCGCACAAC	Quantification of *cc9* expression by q(RT)PCR
O8	PR1-qRT-rv	ZMU82200	CGGAGTGGATCAGCTTGCAGTC	
O9	ATFP4-qRT-fw	NP_001152411.1	CAGCCTGTGGACATATGC	Quantification of *atfp4* expression by q(RT)PCR
O10	ATFP4-qRT-rv	NP_001152411.1	GCACATGCCCTTAACCTC	
O11	GAPDH-qRT-fw	NM001111943	CTTCGGCATTGTTGAGGGTTTG	normalizer gene for q(RT)PCR
O12	GAPDH-qRT-rv	NM001111943	TCCTTGGCTGAGGGTCCGTC	

pB3-3 Primers

#	Name	Reference	Sequence 5'→3'	Application
O13	POX12-Frag1-pB3-3-fw	EU964425.1	CCCAAGCTTTGCTGTCCTTCCTGGCGA	Amplification of *pox12* fragment2 for cloning in pB3-3
O14	POX12 Frag1 pB3-3 rv	EU964425.1	CCCAAGCTTCACGTAGTACTTGTTGTC	
O15	POX12-Frag2-pB3-3-fw	EU964425.1	CCCAAGCTTACGCCCAACGCCTTCGAC	Amplification of *pox12* fragment2 for cloning in pB3-3
O16	POX12-Frag2-pB3-3-rv	EU964425.1	CCCAAGCTTCCATCTTGACGTAGGAGT	
O17	YFP-fw	p123-yfp (Weber *et al.*, 2003)	CCCAAGCTTTGGTGCCCATCCTG	Amplification of *yfp* fragment for cloning in pB3-3
O18	YFP-rv	p123-yfp (Weber *et al.*, 2003)	CCCAAGCTTAGCCGAAGGTGGTC	

pET15b Primers

#	Name	Reference	Sequence 5'→3'	Application
O19	NdeI_Pep1-fw	XP_758134	TTCATATGGATGCTGCGGGTGCGGTACCATTG	Amplification of *pep1* for cloning in pET15b
O20	Pep1_BamHI-rv	XP_758134	TTGGATCCGGATGCTGCGGGTGCGGTACCATTG	
O21	NdeI_RSIATA-GFP-fw	p123 (Aichinger et al., 2003)	GGAATTCCATATGCGCTCGATCGCCACCGCCAT	Amplification of GFP for cloning in pET15b
O22	GFP_BamHI-rv	p123 (Aichinger et al., 2003)	CGCGGATCCTTACTTGTACAGCTCGTC	

BiFC Primers

#	Name	Reference	Sequence 5'→3'	Application
O23	XhoI_RSIATA-CFP-fw	EU530627.1	CTCGAGCGCTCGATCGCCACCGCCGTGAGCAAGGGCGAGGAGCTGTTC	Amplification of CFP for cloning into BiFC vectors
O24	CFP_XmaI-rv	EU530627.1	CCCGGGCTTGTACAGCTCGTCCATGCC	Amplification of CFP and mCherry for cloning into BiFC vectors
O25	XhoI_RSIATA-cherry-fw	(Doehlemann et. al., 2009)	CTCGAGCGCTCGATCGCCACCGCCGTGAGCAAGGGCGAGGAGGATAAC	Amplification of mCherry for cloning into BiFC vectors
O26	NdeI_BiFCinv-LB-fw	(Waadt & Kudla, 2008)	GGAATTCCATATGTTAGCTTGCATGCCTGCAGGTCC	Amplification of BiFC vector to remove MCS
O27	BiFCinv-LB-rv_HindIII	(Waadt & Kudla, 2008)	GGGAAGCTTCGTTACACCACAATATATCCTGCCAAGATCTCTAATTCCGGGG	
O28	NdeI_BiFCinv-Tnos-fw	(Waadt & Kudla, 2008)	GGAATTCCATATGGGGGATAACGCAGGAAAGAACATGAAGGCC	
O29	BiFCinv-Tnos _HindIII-rv	(Waadt & Kudla, 2008)	GGGAAGCTTCCCGATCTAGTAACATAGATGACACCGCGCGC	
O30	BamH1_pPep1-fw	XM_753041.1, codon optimized by GenScript, Piscataway, USA for expression in plant	CGCGGATCCAATGTCCAAGCCCTTCCTCTC	Amplification of codon optimized Pep1 for cloning into BiFC vectors
O31	pPep1_XhoI-rv	XM_753041.1, codon optimized by GenScript, Piscataway, USA for expression in plant	CCGCTCGAGAATGGCGTAATCAGGGACATCGTAAGG	
O32	BamHI_POX12-fw	EU964425.1	CGCGGATCCATGGCGGCTGCTACTTCTTC	Amplification of POX12 for cloning into BiFC vectors
O33	POX12_XhoI-rv	(Waadt & Kudla, 2008)	CTTCTCGAGTAGCACGAGGCTACCGGC	
O34	EcoRI_SPYNE-fw	(Waadt & Kudla, 2008)	CGAATTCATCTCGAGCGCTCGATCGCCAC	Amplification of BiFC vectors for cloning control constructs
O35	ViciaSP_EcoRI-rv	(Waadt & Kudla, 2008)	GGAATTCTAGCAAGGCAGGTGGAAGTGAAG	

Yeast-Two-Hybrid Primers

O36	XmaI-Pep1-fw	XP_758134	CTATCCCGGGTATGGATGCTGCGGGTGCGGTACC	Amplification of Pep1 for cloning into pGADT7
O37	Pep1-XhoI-rv	XP_758134	CCGCTCGAGTTACATGCCAAACATGCTACC	
O38	NdeI-POX12-fw	EU964425.1	GATCACATATGATGGCCGAGGGGGCCAGGTAC	Amplification of POX12 for cloning into pGBKT7
O39	POX12-EcoRI-rv	EU964425.1	CTCGAATTCCTAGAGCACGAGGCTACC	
O40	POX12_Mut1	EU964425.1	CGCGGCGCTGATCGGGCTCCACGTCCTCGACTGCTTCGTG	Introduction of point mutations to the active site of POX12
O41	POX12_Mut2	EU964425.1	TGTCGGGCGGGCTCACGGTGGGCATC	
