# Supplementary material for: The Ustilago maydis Effector Pep1 Suppresses Plant Immunity by Inhibition of Host Peroxidase Activity
Source: PLoS Pathog. 2012 May 10;8(5):e1002684. doi: 10.1371/journal.ppat.1002684 (PMC3349748; doi:10.1371/journal.ppat.1002684)
Supplement: Table S2 — Relative expression of pox12 in response to H2O2. (PDF) [file ppat.1002684.s009.pdf]

Table S2 Relative expression of *pox12* in response to H_2O_2

Relative expression of *pox12* in response to H_2O_2 infiltration was determined by qRT-PCR. Expression levels are relative to water treated plants. Data represent two biological replicates with two technical replicates each. P values were calculated by an unpaired t-test.

**Sample**	**Relative Expression**	**Standard Deviation**	**P value**
1 mM H_2O_2, 2 hours*	0.734	0.241	0.422
10 mM H_2O_2, 2 hours*	0.826	0.399	0.348
1 mM H_2O_2, 4 hours*	1.003	0.388	0.997
10 mM H_2O_2, 4 hours*	1.218	0.214	0.572

* time after H_2O_2 treatment
